# Formation of Proton Motive Force Under Low-Aeration Alkaline Conditions in Alkaliphilic Bacteria

**DOI:** 10.3389/fmicb.2018.02331

**Published:** 2018-10-02

**Authors:** Toshihide Matsuno, Toshitaka Goto, Shinichi Ogami, Hajime Morimoto, Koji Yamazaki, Norio Inoue, Hidetoshi Matsuyama, Kazuaki Yoshimune, Isao Yumoto

**Affiliations:** ^1^Department of Chemistry and Biology, National Institute of Technology, Fukui College, Sabae, Japan; ^2^Bioproduction Research Institute, National Institute of Advanced Industrial Science and Technology (AIST), Sapporo, Japan; ^3^Graduate School of Agriculture, Hokkaido University, Sapporo, Japan; ^4^Department of Bioscience and Technology, School of Biological Sciences and Engineering, Tokai University, Sapporo, Japan; ^5^Division of Marine Life Science, Faculty of Fisheries Sciences, Hokkaido University, Hakodate, Japan; ^6^Hakodate Junior College, Hakodate, Japan; ^7^College of Industrial Technology, Nihon University, Narashino, Japan

**Keywords:** alkaliphilic, bioenergetic mechanism, cytochrome *c*, membrane electrical potential, Donnan effect, proton condenser, *Bacillus*, *Pseudomonas*

## Abstract

In Mitchell’s chemiosmotic theory, a proton (H^+^) motive force across the membrane (Δp), generated by the respiratory chain, drives F_1_F_o_-ATPase for ATP production in various organisms. The bulk-base chemiosmotic theory cannot account for ATP production in alkaliphilic bacteria. However, alkaliphiles thrive in environments with a H^+^ concentrations that are one-thousandth (ca. pH 10) the concentration required by neutralophiles. This situation is similar to the production of electricity by hydroelectric turbines under conditions of very limited water. Alkaliphiles manage their metabolism via various strategies involving the cell wall structure, solute transport systems and molecular mechanisms on the outer surface membrane. Our experimental results indicate that efficient ATP production in alkaliphilic *Bacillus* spp. is attributable to a high membrane electrical potential (ΔΨ) generated for an attractive force for H^+^ on the outer surface membrane. In addition, the enhanced F_1_F_o_-ATPase driving force per H^+^ is derived from the high ΔΨ. However, it is difficult to explain the reasons for high ΔΨ formation based on the respiratory rate. The Donnan effect (which is observed when charged particles that are unable to pass through a semipermeable membrane create an uneven electrical charge) likely contributes to the formation of the high ΔΨ because the intracellular negative ion capacities of alkaliphiles are much higher than those of neutralophiles. There are several variations in the adaptation to alkaline environments by bacteria. However, it could be difficult to utilize high ΔΨ in the low aeration condition due to the low activity of respiration. To explain the efficient ATP production occurring in H^+^-less and air-limited environments in alkaliphilic bacteria, we propose a cytochrome *c*-associated “H^+^ capacitor mechanism” as an alkaline adaptation strategy. As an outer surface protein, cytochrome *c*-550 from *Bacillus* *clarkii* possesses an extra Asn-rich segment between the region anchored to the membrane and the main body of the cytochrome *c*. This structure may contribute to the formation of the proton-binding network to transfer H^+^ at the outer surface membrane in obligate alkaliphiles. The H^+^ capacitor mechanism is further enhanced under low-aeration conditions in both alkaliphilic *Bacillus* spp. and the Gram-negative alkaliphile *Pseudomonas alcaliphila*.

## Introduction

Bacteria that thrive under extreme environmental conditions, such as low or high temperatures and high or low pH, are called extremophiles. Each extremophile possesses certain strategies for adaptation under different conditions. These complex strategies consist of environmental adaptation mechanisms, such as alterations in specific characteristics of enzymes and membranes. Although there is a common fundamental basis for extremophile metabolism, these organisms differ slightly in the characteristics of certain components necessary for environmental adaptation (e.g., modification of cell surface structures and protein characteristics and production of molecular chaperones).

Alkaliphiles are defined as microorganisms that exhibit better growth at pH ≥ 9 than at pH < 9. Since Vedder (1934) isolated the obligate alkaliphile *Bacillus alcalophilus*, many alkaliphilic *Bacillus* strains have been isolated from common environments such as garden soil and horse manure in the search for enzymatic resources, as alkaliphilic *Bacillus* strains produce heat-stable enzymes (Horikoshi and Akiba, 1982; Horikoshi and Grant, 1998; Horikoshi, 2006, 2011). Although various strains have been isolated, until Nielsen et al. (1995) proposed nine new *Bacillus* spp., it was not known whether alkaliphilic bacteria comprise multiple species due to the lack of a molecular identification method concomitant with a gene sequence database. Since the discovery made by Nielsen et al. (1995) many additional alkaliphilic bacteria have been isolated and identified as new species. Related reports indicate that diverse alkaliphiles are distributed in a variety of environments, which may indicate the presence of numerous small alkaline niches [e.g., the termite gut (Thongaram et al., 2003)] and/or large high-pH environments (e.g., alkaline lakes). Alkaline environments have been present throughout Earth’s history. Zavarzin (1993) hypothesized that modern soda lakes may represent a refuge for relict terrestrial communities from ancient continents of the Early Proterozoic Eon. In contrast, a leading hypothesis suggests that the origin of life can be track back to ocean-floor-based alkaline hydrothermal vents (Lane and Martin, 2012). Natural H^+^ gradients across the membranes of iron monosulfide bubbles could lead to the formation of protocells (Russell and Hall, 1997).

The phylogenetic diversity and wide distribution of alkaliphiles on Earth indicates that the evolution of alkaliphiles is not a specific phenomenon but a common event in natural environments. Therefore, many variations in alkaline adaptation mechanisms should be present in alkaliphiles. The range of reported alkaline adaptation mechanisms has not been explained to date. However, diverse adaptation mechanisms involving secondary cell wall variations were reported by Aono and Horikoshi (1983), Aono et al. (1993, 1995,1999). The formation of a negatively charged secondary cell wall results in the pH at the outer surface membrane being lower than the extracellular pH (i.e., medium pH) (Tsujii, 2002). The diversity of environmental adaptation mechanisms underlying the survival of numerous alkaliphiles of different taxa provides various possible explanations for the existence of life in alkaline environments. Alkaliphilic *Bacillus* spp. have an acidic secondary cell wall. *B. halodurans* C-125 produces an acidic secondary cell walls consisting of teichurono-peptide and teichuronic acid. In contrast, *B.* *pseudofirmus* OF4 cells produces the cell surface protein SlpA and polyglutamic acid (Gilmore et al., 2000). These structures attract H^+^ and repel OH^-^, and the structural functions of those components protects the intracellular metabolic pathways from severe extracellular environments. Although the acidic secondary cell wall is indispensable for alkaline adaptation in alkaliphilic *Bacillus* spp., the variations in the secondary cell wall structure, which are shared among alkaliphilic *Bacillus* spp., have not been elucidated to date.

Alkaline environments are not always favorable for alkaliphilic bacteria. Alkaliphilic *Bacillus* spp. have been reported to produce acid to reduce the pH when the ambient pH is too high for metabolism (Horikoshi, 2006). This acid production can often be observed even in media lacking sugars. In contrast, these bacteria create an alkaline environment when the ambient pH is too low for metabolism. These phenomena indicate that several alkaliphiles have the ability to increase the favorability of the ambient environment. *Amphibacillus iburiensis* is able to grow in broth medium adjusted to pH 11 prior to inoculation (Hirota et al., 2013). However, this bacterium exhibits distinct growth initiation at pH 9 after lowering the pH of the medium via acid production, as observed by monitoring the change in pH during incubation.

Alkaliphiles adapt to the environment by employing various combinations of mechanisms to adjust to alkaline conditions. Alkaliphilic *Bacillus* strains have been reported to adjust the intracellular pH to an appropriate level via the Na^+^/H^+^ antiporter, Na^+^ channels or stator force generator that drives Na^+^-dependent motility (Kitada et al., 1982; Ito et al., 2004a,b; Padan et al., 2005), which contribute to replace the H^+^-potential base transport system with the Na^+^-potential base transport system. Thus, the Na^+^/H^+^ antiporter and other Na^+^ or K^+^-related transport systems reduce the utilization of H^+^ by transport systems, which is very important for alkaliphiles that thrive at one-thousandth the concentration of H^+^ found under neutral conditions. In addition, the rotational torque of the flagella of alkaliphilic *Bacillus* strains is produced by the influx of Na^+^ derived from the Na^+^-base potential across the membrane (Ito et al., 2004a).

It is reasonable to consider that the entire solute transport system functions via the Na^+^ potential across the membrane. However, the ATP synthase-based energy production system is derived from the H^+^-base potential across the membrane (Dimroth and Cook, 2004), which may indicate localization of the H^+^-base potential across the membrane in the vicinity of the respiratory chain in the horizontal direction. In addition, if H^+^ is not attracted to the interface in the vicinity of the outer surface membrane, the H^+^ concentration will be quite low. The H^+^-base potential may also be present in the vicinity of the outer surface membrane in the perpendicular direction.

Alkaliphilic *Bacillus* spp. have been reported to exhibit higher membrane electrical potentials (ΔΨ) than neutralophilic *Bacillus* strains (Yumoto, 2003; Goto et al., 2005). However, the calculation of bulk-base parameters [ΔΨ and transmembrane pH gradient (ΔpH)] for the Δp driving F_1_F_o_-ATPase does not account for ATP production because the high deficiency in ΔpH is not compensated by ΔΨ. We demonstrated the importance of a large ΔΨ in alkaliphiles by showing the contribution of ΔΨ to the retention of H^+^ in the vicinity of the outer surface of the membrane in the vertical direction and the contribution of efficient ATP production under conditions involving H^+^ scarcity (Yoshimune et al., 2010). Furthermore, ΔΨ ensures efficient ATP production to enhance the F_1_F_o_-ATPase driving force per H^+^. In addition, structural and physicochemical mechanisms to retain H^+^ at the outer surface of the membrane are indispensable. However, it could be difficult to utilize high ΔΨ in the low aeration condition due to the low activity of respiratory system. In this context, we highlight “a high-potential H^+^ capacitor mechanism” based on the existence of membrane-bound or periplasmic cytochrome *c* in alkaliphiles.

## Background on the General Bioenergetics and Growth of Alkaliphilic *Bacillus* spp.

According to Peter Mitchell’s chemiosmotic theory (Mitchell, 1961), the Δp across the membrane that drives F_1_F_o_-ATPase to produce ATP consists of ΔpH (intracellular pH minus extracellular pH) and the difference in electrical potential across the membrane, i.e., ΔΨ (intracellulary electronegative and extracellularly electropositive across the membrane). The Δp can be calculated by the following formula.

(1) $$\Delta p=\Delta \Psi -Z\Delta pHZ=2.3RT/F=ca.59\;mV(at25^{\circ }C)$$

R = gas constant (8.315 J mol^-1^); T = absolute temperature (298 *K* = 25°C); F = Faraday constant (96.485 kj [v⋅mol]^-1^).

The H^+^ gradient is also utilized for other energy-requiring processes such as transmembrane solute transport and signaling. In general, the parameters Δp, ΔΨ, and ΔpH apply to the bulk-base. Although it is difficult to estimate the values of ΔΨ and ΔpH based on the vicinity of the membrane surface, localization of these parameters in the vicinity of the membrane surface in both the horizontal and vertical directions should be considered, especially in the case of the outer surface membrane of alkaliphilic *Bacillus* spp. (Gennis, 2016; Sjöholm et al., 2017). These characteristics are common to biological energy production mechanisms. Alkaliphilic bacteria must possess distinctive characteristics at the outer surfaces of the membrane to effectively utilize existing stores of H^+^ at the outer surface membranes.

Although bulk-base calculations cannot account for ATP production by alkaliphilic *Bacillus* spp. Due to the great hindrance conferred by negative ΔpH values, the growth of these bacteria is vigorous under alkaline conditions (Guffanti and Hicks, 1991). The growth features of facultative alkaliphilic *B*. *pseudofirmus* OF4 were estimated at a steady state under various pH-controlled culture conditions. Strain OF4 exhibits specific growth rates of 0.77 and 1.10 h^-1^ in media maintained at pH values of 7.5 and 10.6, respectively. We estimated the growth characteristics of the obligate alkaliphilic *B*. *clarkii* K24-1U in batch culture. Strain K24-1U exhibited more rapid growth (μ_max_ = 0.33 h^-1^) than the neutralophilic *B*. *subtilis* IAM 1026 (μ_max_ = 0.26 h^-1^) (Hijikata, 2004). These superior growth characteristics are attributable to vigorous ATP production. Thus, in alkaliphilic *Bacillus* spp., these growth characteristics are not accounted for by bulk-based bioenergetic parameters.

## Background on Proton Behavior at the Outer Surface of the Membrane

The zone formed in the vicinity of the outer surface of the membrane formed due to the presence of phosphate and carbonyl headgroups stabilizes excess hydrated H^+^ relative to the bulk solution. However, the pH of the bulk phase at the outer side membrane in alkaliphiles is expected to be at least 2 units higher than that in neutralophiles. In addition, the rate of H^+^ exchange between the deep interface zone in the vicinity of the outer surface membrane and the bulk phase in alkaliphiles may be greater than that in neutralophiles due to the large difference in pH between the deep interface zone and the bulk phase at the outer membrane (Mulkidjanian et al., 2006; Gennis, 2016). Therefore, it survival under proton-less conditions without specific alkaline adaptation mechanisms is difficult. Although many neutralophilic *Bacillus* spp. can grow at pH 9, most cannot grow well at pH 10, unlike alkaliphilic *Bacillus* spp.

The H^+^ transfer mechanism on the outer surface of the membrane is dependent on the distance between the H^+^-vent (e.g., a respiratory complex such as cytochrome *c* oxidase) and H^+^-sink (e.g., F_1_F_o_-ATPase). Sjöholm et al. (2017) investigated the distance effect between the terminal oxidase, cytochrome *bo*_3_ and F_1_F_o_-ATPase by reconstituting these proteins in a vesicle and measuring ATP synthesis activity. An even shorter distance between the H^+^-vent and H^+^-sink than that observed in neutralophiles was expected to be favorable in alkaliphiles. A high cytochrome content has been reported in alkaliphilic *Bacillus* spp. (Lewis et al., 1980; Guffanti et al., 1986; Yumoto et al., 1997; Goto et al., 2005). This feature will relate to H^+^ behavior at the outer surface membrane.

## Proton Behavior at the Outer Surface Membrane in Alkaliphiles

It is considered that the H^+^ concentration at the outer surface membrane in alkaliphiles is lower than that in neutralophiles due to the low background H^+^ concentration in alkaliphiles. In addition, the rate constant of H^+^ exchange between the deep interface zone of the outer surface membrane and the adjacent bulk zone may be higher in alkaliphiles than in neutralophiles due to the larger pH gap between the deep interface zone of the outer surface membrane and the adjacent bulk zone in alkaliphiles than that in neutralophiles. In addition to the membrane-surface-based ΔpH (ΔpH values) (Xiong et al., 2010), membrane-surface-based ΔΨ values may also exist (Lyu and Lazár, 2017). In addition, proteins present on the outer surface membrane may play a role in the retention of H^+^ in this region. A comparative experiment to detect H^+^ in the bulk phase during respiration in the obligate alkaliphilic *B*. *clarkii* K24-1U and the neutralophilic *B*. *subtilis* IAM 1026 was performed by monitoring the change in pH (Yoshimune et al., 2010). Whole-cell suspensions of both *Bacillus* stains consumed oxygen immediately after the introduction of oxygen, and a lag period was observed for H^+^ extrusion by the respiratory chain into the bulk phase. The lag period for alkaliphilic *B*. *clarkii* K24-1U was significantly longer at pH 10 than at other pH values and significantly longer than those for *B*. *subtilis* at various pH values. This observation may indicate that H^+^ was transferred into the bulk phase after all the H^+^ retention sites were occupied by H^+^ translocated via respiratory complexes. The introduction of monensin, which exchanges extracellular H^+^ for intracellular Na^+^, similar to a Na^+^/H^+^ antiporter, resulted in a prolonged lag period for H^+^ extrusion to the bulk, indicating that the H^+^ present at H^+^ retention sites on the outer surface membrane translocated to the intracellular side via countertransport with the Na^+^ present in the intracellular space. In contrast, when a ΔΨ-disrupting agent such as valinomycin or ETH-157 was introduced, the lag phase disappeared. These experiments reveal the meaning of ΔΨ values in alkaliphilic *Bacillus* spp. are larger than those in neutralophilic *Bacillus* spp.

## Efficiency of H^+^ Translocation From the Outer Surface Membrane to F_1_F_o_-Atpase

The growth of alkaliphilic *Bacillus* spp. is much faster than that of neutralophilic *Bacillus* spp. as described above, which may be attributable to the higher ATP production rate in alkaliphilic *Bacillus* spp. than that in neutralophilic *Bacillus* spp. The ATP production rate was estimated using the obligate alkaliphile *B*. *clarkii* DSM 8720^T^ and neutralophilic *B*. *subtilis* IAM 1026 (Hirabayashi et al., 2012). *B*. *clarkii* DSM 8720^T^ produced 7.2 nmol ATP⋅mg protein^-1^⋅min^-1^ (endogenous substrate) at pH 10, which was comparable to the amount produced by *B*. *pseudofirmus* OF4 (6.6 ± 3.9 nmol ATP⋅mg protein^-1^⋅min^-1^ [starved cells re-energized with malate]) at pH 10.5 (Guffanti and Krulwich, 1992). In contrast, *B*. *subtilis* IAM 1026 produced 0.96 nmol ATP⋅mg protein^-1^⋅min^-1^ at pH 7. Thus, the ATP production rate in alkaliphilic *Bacillus* spp. was much higher (6.9-7.5 times) than that in neutralophilic *B*. *subtilis*.

If rapid ATP production by alkaliphilic *Bacillus* spp. was attributable to rapid H^+^ translocation across the membrane by respiratory complexes, then these bacteria would exhibit a rapid O_2_ consumption and/or high efficiency of H^+^ translocation across the membrane per molecule of O_2_ consumed. However, the oxygen consumption rate of *B*. *clarkii* DSM 8720^T^ cells was 0.19 μmol O_2_⋅min^-1^⋅mg cell protein^-1^ at pH 10. Although these data were obtained with an endogenous substrate, the results were comparable to those obtained using malate as the substrate in other alkaliphilic *Bacillus* spp. (Lewis et al., 1980; Guffanti et al., 1986; Aono et al., 1996). The oxygen consumption rate of *B*. *subtilis* IAM 1026 was 0.50 μmol O_2_⋅min^-1^⋅mg cell protein^-1^ at pH 7. Thus, the oxygen consumption rate of whole *B*. *subtilis* IAM 1026 cells at pH 7 was 2.6 times higher than that of *B*. *clarkii* DSM 8720^T^ at pH 10. Unlike the theoretical H^+^/O ratio calculated for *Bacillus* spp. (complex III, 4 plus complex IV, 2 = 6) (Sone et al., 1999), the H^+^/O ratio in *B*. *clarkii* DSM 8720^T^ was 3.6 at pH 10. In contrast, the H^+^/O ratio in *B*. *subtilis* IAM 1026 was 5.3. The H^+^/O ratio in *B*. *subtilis* IAM 1026 was 1.5 times higher than that in *B*. *clarkii* DSM 8720^T^ (Goto et al., 2016). Considering both the O_2_ consumption rate and the H^+^/O ratio in both strains, the H^+^ translocation rate in *B*. *subtilis* IAM 1026 (pH 7) was 3.9 times higher than that in *B*. *clarkii* DSM 8720^T^ (pH 10). This finding indicated that the higher ATP production rate in neutralophilic *B*. *subtilis* IAM 1026 than that in alkaliphilic *B*. *clarkii* DSM 8720^T^ cannot be accounted for by the rate of H^+^ translocation across the membrane via the respiratory chain. The large differences in ATP production rates between *B*. *clarkii* DSM 8720^T^ and *B*. *subtilis* IAM 1026 are attributable to differences in the F_1_F_o_-ATPase driving force per H^+^, which may arise from differences in ΔΨ. It is difficult to translocate H^+^ from the intracellular to the extracellular side of the membrane because ΔΨ hinders translocation in that direction by electrical attraction. However, once translocated to the outer surface of the membrane, H^+^ has high potential to drive F_1_F_o_-ATPase. It has been reported that the ΔΨ in alkaliphilic *Bacillus* spp. is larger than that in neutralophilic *Bacillus* spp. (Hoffmann and Dimroth, 1991; Krulwich, 1995; Yumoto, 2002; Goto et al., 2005; Hirabayashi et al., 2012). The same phenomenon has been observed in the alkaliphilic *B*. *clarkii* DSM 8720^T^ and neutralophilic *B*. *subtilis* IAM 1026. The ΔΨ values of *B*. *clarkii* DSM 8720^T^ and *B*. *subtilis* IAM 1026 were -192 mV at pH 10 and -122 mV at pH 7, respectively (Goto et al., 2016).

## Occurrence of ΔΨ

As described above, ΔΨ is very important for attracting H^+^, which translocates via the respiratory chain to the outer surface membrane. Generally, the ΔΨ across the membrane is generated by translocation of positively charged H^+^ from the intracellular to the extracellular region across the membrane. However, the larger ΔΨ in alkaliphilic *Bacillus* spp. than that in neutralophilic *Bacillus* spp. cannot be accounted for by the rate of H^+^ translocation. The large ΔΨ values of alkaliphilic *Bacillus* spp. may be attributed to the production of high Donnan potentials (Donnan, 1924). The Donnan effect is observed in the presence of membrane-impermeable charged molecules on only one side of the membrane. If a membrane-impermeable negatively charged molecules are present only on the intracellular side of the membrane, these molecules contribute to the formation of the ΔΨ. To estimate the contribution of the Donnan effect to the large ΔΨ in alkaliphilic *Bacillus* spp., intracellular negative ion capacity was estimated in the obligate alkaliphilic *B. clarkii* DSM 8720^T^ and facultative alkaliphilic *B. cohnii* YN-2000 and compared to that of the neutralophilic *B*. *subtilis* IAM 1026. To estimate the intracellular negative ion capacity, a cell extract containing inside-out membrane vesicles was prepared and titrated using the positively charged substance clupein sulfate. Negative ion capacity is the amount of negative surface charge of substances in a solution (substances in the intracellular fraction of bacterial cells in this study) estimated by the colloid titration method. This method is one procedure by which the net charge density of surfaces, polyelectrolytes levels, and charge demand of colloidal materials in a solution may be estimated. The measured parameter is the capacity of the mixture to adsorb a polyelectrolyte with the opposite net charge. The intracellular negative ion capacity in alkaliphilic *Bacillus* spp. increased with increasing pH in the range of pH 6-8, whereas the intracellular negative ion capacity of neutralophilic *B*. *subtilis* IAM 1026 changed very little within this pH range. To understand the corresponding negative ion capacity when alkaliphilic *Bacillus* spp. were grown under alkaline conditions, the intracellular pH of the alkaliphilic strains was estimated at an extracellular pH of 10 (Goto et al., 2016). The intercellular pH of the alkaliphilic *Bacillus* spp. *B*. *clarkii* DSM 8720^T^ and *B*. *cohnii* YN-2000 was 8.1, which is comparable to values reported previously using other alkaliphilic *Bacillus* spp. (Guffanti et al., 1986; Guffanti and Hicks, 1991). At this intracellular pH, the negative ion capacities of *B*. *clarkii* DSM 8720^T^ and *B*. *cohnii* YN-2000 were 2.9 and 3.3 (×10^6^ eq⋅mg protein^-1^), respectively. The intracellular pH of neutralophilic *B*. *subtilis* IAM 1026 was 6.7 when the extracellular pH was 7. At this intracellular pH, the negative ion capacity of *B*. *subtilis* IAM 1026 was 0.7 (×10^6^ eqmg protein^-1^). This finding indicates that alkaliphilic *Bacillus* spp. possess a much higher intracellular negative ion capacity than neutralophilic *B*. *subtilis*. This high intracellular negative ion capacity contributes to the intrinsic ΔΨ. There may be questions regarding the existence of such a high intracellular negative ion capacity. One explanation is that the intracellularly expressed acidic proteins in alkaliphilic *Bacillus* spp. are negatively charged at slightly higher intercellular pH values (ca. pH 8) than those in neutralophiles (pH 6-7). Whole-genome analyses have been performed previously in alkaliphilic *Bacillus* spp. as well as neutralophilic *Bacillus* spp., and Janto et al. (2011) estimated the average p*I* values of proteins localized in intracellular and extracellular spaces, the cell wall and the membrane. Despite a tendency toward a high frequency of acidic proteins in the cell wall and extracellular proteins in alkaliphiles, the obligate alkaliphile *B. selenitireducens* ML10 possesses much higher levels of low p*I* proteins than neutralophiles. The combination of the acidic nature of the intracellular side chains of acidic membrane proteins and intercellular acidic proteins is predicted to contribute to the high negative ion capacity in alkaliphilic *Bacillus* spp.

## Cytochromes *c* From Various *Bacillus* spp. and Related Taxa

The primary role of cytochrome *c* is to transfer electrons between complex III (cytochrome *bc*_1_ complex) and complex IV (cytochrome *c* oxidase) proteins. A typical class I cytochrome *c* possesses one low-spin heme *c* in the N-terminal region bound to the protein by two thioether bonds with cysteine residues (Ambler, 1991). The proximal side and distal side of the iron ligands have a histidine residue and a methionine residue, respectively. The molecular weight of cytochrome *c* is approximately 8,000-14,000, and cytochrome *c* is a soluble protein in mitochondria and Gram-negative bacteria (Pettigrew and Moore, 1987; Moore and Pettigrew, 1990; Yamanaka, 1992; Brayer and Murphy, 1996).

Although numerous studies have assessed soluble cytochromes *c* from various sources, including mitochondria and Gram-negative bacteria, there have been limited examples of cytochrome *c* from Gram-positive bacteria. Despite having some outer surface membrane space, Gram-positive bacteria do not have outer membranes similar to those of Gram-negative bacteria. Therefore, Gram-positive bacteria do not possess a periplasmic space equivalent to that in Gram-negative bacteria. Consequently, all the cytochrome *c* in Gram-positive bacteria is membrane-bound. For example, *B*. *subtilis* possesses two types of membrane-bound cytochromes: *c*-550 and *c*-551. Cytochrome *c*-550 has a molecular mass of 13 kDa, with a membrane-anchored domain consisting of a single α-helical transmembrane segment of a hydrophobic polypeptide containing 30 amino acids (von Wachenfeldt and Hederstedt, 1990). The midpoint redox potential of cytochrome *c*-550 is +178 mV (von Wachenfeldt and Hederstedt, 1993). The other cytochrome *c*, namely, *c*-551, has a molecular mass of 10 kDa and binds to the membrane via a diacyl-glyceryl-cysteine moiety (Bengtsson et al., 1999). The midpoint redox potential of cytochrome *c*-551 is >100 mV. The functions of these two membrane-binding cytochromes *c* have not been characterized. However, it is expected that these proteins are involved in transport of electrons between complexes III and IV.

It has been reported that alkaliphilic *Bacillus* spp. contain higher amounts of membrane-bound cytochrome *c* than neutralophilic *B*. *subtilis* (Yumoto et al., 1997; Hijikata, 2004). In addition, the amount of membrane-bound cytochrome *c* is low in mutant strains that lack the ability to grow in alkaline media (Lewis et al., 1980). These findings suggest that membrane-bound cytochrome *c* in alkaliphiles may contribute to adaptation in alkaline environments. Cytochrome *c*-552 from *B. pseudofirmus* RAB was first purified and characterized from alkaliphilic *Bacillus* spp., exhibiting a molecular mass of 16.5 kDa and midpoint redox potential of +66 mV at pH 7, which decreases as the surrounding pH is increased (Davidson et al., 1988). This cytochrome *c* is normally membrane bound but was purified as a soluble protein. Cytochrome *c* proteins are predicted to play a role in electron transport between complexes III and IV.

The primary and 3D structures of cytochrome *c* were first studied in alkaliphilic *Bacillus*-related taxa using cytochrome *c*-553 purified from *Sporosarcina pasteurii* (formerly *B. pasteurii*) (Benini et al., 2000). Cytochrome *c*-553 has a low molecular mass of 9.6 kDa and a low midpoint redox potential (+47 mV) (Benini et al., 1998). The crystal structure of the protein exhibits a highly asymmetric charge distribution. Most of the charges are located on the side opposite to that exposed to the heme edge. The localization of charges is related to H^+^ transfer on the outer surface membrane. Analyses of the physicochemical parameters reveal that the heme solvent accessibility is correlated with entropy. This finding suggests a direct link between the major determinant of the electrochemical potential (entropy) and a structural parameter (heme solvent exposure). The low midpoint redox potential of cytochrome *c*-553 could be explained by the decrease in reduction entropy via extrusion of water molecules from the protein hydration shell, affecting a large number of water molecules in the case of increased solvent accessibility, which occurs upon heme reduction.

The abundance of alkaliphilic *Bacillus* spp.-specific membrane-bound cytochrome *c* (Yumoto et al., 1991) has also been studied using the facultative alkaliphile *B. cohnii* YN-2000 (Yumoto et al., 2000). The abundance of membrane-bound cytochrome *c* is higher during growth at pH 10 than during growth at pH 7. In addition, cytochrome abundance is further increased under low-aeration conditions. Solubilized *B*. *cohnii* YN-2000 membranes prepared from cells grown at pH 10 using the detergent Triton X-100 contain larger amounts of cytochrome *c*-553 than solubilized membranes prepared with cells grown at pH 7. The native molecular mass of cytochrome *c*-553, which was solubilized by Triton X-100 in a buffer, was determined to be 127 kDa by gel filtration. The molecular mass of Triton X-100, which was used to solubilize cytochrome *c*-553 in solution is 90 kDa. Therefore, if cytochrome *c*-553 is contained in micelles of Triton X-100, the actual native molecular mass should be 37 kDa. The stoichiometry of cytochrome c-553 and Triton X-100 is considered to be 1:1. In contrast, the molecular mass of cytochrome *c*-553 was determined to be 10,500 Da by sodium dodecyl sulfate-polyacrylamide gel electrophoresis (SDS-PAGE). The results described above suggest that cytochrome *c*-553 forms a tetramer in its native form in solution or in the original membrane. Cytochrome *c*-553 from *B*. *cohnii* YN-2000 also exhibits a low midpoint redox potential of +87 mV at the pH values ranging from 6 to 8.

A novel type of cytochrome *c* oxidase, cytochrome *aco*_3_, was purified and characterized from *B*. *cohnii* YN-2000 (Qureshi et al., 1990; Yumoto et al., 1993; Denda et al., 2001). Cytochrome *c*-533 can react with cytochrome *aco*_3_, and the reaction is greatly enhanced in the presence of the positively charged substance poly-L-lysine, which may accelerate binding to two negatively charged molecules: cytochrome *c*-553 and cytochrome *aco*_3_. Although cytochrome *aco*_3_ was present in equal amounts in cells grown at pH 10 and pH 7, the cytochrome *c*-553 content was higher in cells grown at pH 10 than in those grown at pH 7. The midpoint redox potential of the attached cytochrome *c* was +95 mV at pH 7 (Orii et al., 1991). In contrast, cytochrome *a* exhibited two forms with midpoint redox potentials of +250 mV and +323 mV at pH 7 (Orii et al., 1991). A stopped-flow study on cytochrome *aco*_3_ showed that the cytochrome *a* component exhibited the highest affinity for electrons and only a minimal contribution to O_2_ reduction among the involved redox components. Therefore, membrane-bound cytochrome *c*-553 may directly react not only with cytochrome *c* in cytochrome *aco*_3_ but also with the cytochrome *a* moiety in cytochrome *aco*_3_. During electron flow from cytochrome *c*-553 or cytochrome *c* to cytochrome *a* in cytochrome *aco*_3_, there is a large midpoint redox potential difference between each component. This significant difference is necessary for electron flow between each cytochrome *c* and cytochrome *a* in cytochrome *aco*_3_ to overcome the large ΔΨ (Yumoto et al., 1993). Therefore, the large difference in redox potential between the outer surface redox components and intramembrane redox components sustains electron transfer in the respiratory system in alkaliphilic *Bacillus* spp. In summary, this large difference in midpoint redox potential may be necessary for the generation of produce the large energy potential required for the translocation of intracellular H^+^ to overcome the hindrance conferred by the high ΔΨ and to retain H^+^ on the intracellular side of the membrane.

## Membrane-Bound Cytochrome *c*-550 From the Obligate Alkaliphile *Bacillus clarkii* K24-1U

A previous study suggested that cytochrome *c* has an important role in the adaptation to alkaline environments based on its abundance (Lewis et al., 1980; Guffanti et al., 1986; Yumoto et al., 1991, 1997). In addition, cytochrome *c* is likely located at the outer surface membrane and associated with H^+^ transfer in the vicinity of the membrane. Therefore, we attempted to elucidate the precise structure of membrane-bound cytochrome *c* in alkaliphilic *Bacillus* spp. An experiment was performed to isolate intact protein from the obligate alkaliphile *B.* *clarkii* K24-1U. *Bacillus* spp. generally exhibit strong protease activity, making it difficult to isolate intact cytochrome *c* from their cells. This strong protease activity often causes isolated cytochrome *c* to exhibit an anchor-less, soluble protein. First, an attempt was made to isolate obligate alkaliphilic *Bacillus* spp. that exhibit low protease activity. Screening was performed using soil samples from approximately 10 sites in the Hokkaido region of Japan. One strain, K24-1U, isolated from Yuubari in Hokkaido Japan (43°04^′^ N, 141°58^′^ E), was found to be an obligate alkaliphile that exhibited very weak protease activity. The cytochrome *c*-550 gene sequence was determined and cloned, and the protein was purified and characterized (Ogami et al., 2009). Purified cytochrome *c*-550 was attached to a diacylglycerol-cysteine moiety. According to the analyzed gene sequence of cytochrome *c*-550, a signal peptide was present at the 5^′^ end of the gene. During the processing of mature cytochrome *c*-550, expressed cytochrome *c*-550 was translocated to the extracellular side of the membrane by the signal peptide. After the signal peptide was dissociated, the terminal cystatin was modified by attaching diacylglycerol and acetyl moieties. Cytochrome *c*-550 binds to fatty acids with carbon lengths of C_15_, C_16_, and C_17_ via glycerol-Cys_18_. Although the length of the internal carbon chain is always C_15_, the external chain length varies from C_15_-C_17_. The diacylglycerol moiety exhibits flexibility in its fatty acid molecular species. Therefore, if the expressed amount of cytochrome *c*-550 is dependent on the culture conditions, the fatty acid composition of the membrane may be minimally influenced.

The amino acid sequence of cytochrome *c*-550 was deduced from the gene sequence and aligned with the amino acid sequences of cytochromes *c* from obligate and facultative alkaliphilic and neutralophilic *Bacillus* spp. and related taxa (Ogami et al., 2009). The results indicated that cytochrome *c*-550 contains only two basic amino acids, including histidine, in the heme *c* axial ligand. This scarcity of basic amino acids is more pronounced in obligate alkaliphilic *Bacillus* spp. than in facultative alkaliphiles and neutralophiles. Cytochrome *c*-550 contains the distinct amino acid sequence Gly_22_-Asn_34_, which is absent in facultative alkaliphilic and neutralophilic *Bacillus* spp. Thus, this sequence exists specifically for adaptation to alkaline environments. The amino acid sequence Gly_22_-Asn_34_ contains the H^+^-transferable amino acids Asp and Glu at ratios of 3/13 and 1/13, respectively. The most prominent constituent of the Gly_22_-Asn_34_ sequence is Asn. Asn is present at a ratio of 7/17 in the Asn_21_-Asn_37_ region and may play an important role in the H^+^ transfer network in the vicinity of the outer surface membrane.

Although Asn is theoretically H^+^-transferable, there have been a few examples of the contribution of H^+^ transfer processes due to weak hydrogen binding of the involved residues. Doukov et al. (2007) reported a very interesting hypothetical model of a hydrogen-bond network involving the H^+^-transferable characteristics of Asn in the methyltetrahydrofolate (MTHF) corrinoid-iron-sulfur protein methyltransferase. The enzyme catalyzes the transfer of the methyl group of MTHF to cob(I)amide. This transfer reaction requires electrophilic activation of the methyl group of MTHF, which includes proton transfer to the N5 group of the pterin ring of MTHF. However, the resolved crystal structure of the methyltransferase revealed no obvious H^+^ donor within hydrogen-bonding distance of the N5 position of MTHF. Combining kinetic and structural evidence, it was predicted that the extended hydrogen-bond network contributes to H^+^ transfer to the N5 group of the pterin ring of MTHF. This extended hydrogen-bonding network contains an Asn, a conserved Asp and a water molecule. The evidence from this study suggests that even amino acid residues that exhibit weak hydrogen bonding, such as Asn can contribute to a cumulative hydrogen-bond network such that the overall effect on this transitional state is greater than expected based on the individual components alone. If this knowledge is applied to the Asn_21_-Asn_37_ region in cytochrome *c*-550 in *B.* *clarkii* K24-1U, then the region contributes to a cumulative hydrogen-bond network.

Based on the structures of other reported membrane-bound cytochromes *c* (David et al., 2000), it can be assumed that the region from Asn_21_ to Asn_37_ is located outside the α-helical domain from the N-terminus to the surrounding heme *c*. Thus, the region is located in the vicinity of the outer surface membrane. In addition, we attempted to elucidate the structure of cytochrome *c*-550 in *B.* *clarkii* K24-1U at the original membrane. Cytochrome *c*-550 with a membrane-anchoring diacylglycerol-cysteine moiety exhibits a tetrameric structure in the presence of the detergent Triton X-100. In addition, a mutant protein containing an N-terminal Cys_18_ to Met mutation in the mature protein also exists as a tetramer in the absence of Triton X-100. This finding indicates that cytochrome *c*-550 may exist as a tetramer on the outer surface membrane. This structure may be important not only for the regulation of the redox reaction involving four redox centers, as in the case of cytochrome *c*_3_ in *Desulfovibrio gigas* (Messias et al., 2006) but also for the formation of the hydrogen-bond network.

## H^+^-Coupling Function of Cytochrome *c*

The above-described H^+^/O ratio in *B*. *clarkii* DSM 8720^T^ (H^+^/O ratio = 3.6) cannot be explained by the conventional understanding of the function of respiratory chain because the theoretical H^+^/O ratio in *Bacillus* spp. should be 6 (complex III, 4 *plus* complex IV, 2 = 6) (Sone et al., 1999). The H^+^/O ratio in *B*. *clarkii* DSM 8720^T^, which is lower than the theoretical value, may be attributable to the high amount of energy required to translocate H^+^ across the membrane under a larger ΔΨ value than those of neutralophiles. Due to the low translocation of H^+^ to the outer surface of the membrane, mechanisms for retaining H^+^ and/or regulating H^+^ behavior on the outer surface membrane are necessary. It is expected that the membrane-bound cytochrome *c* concomitant with physicochemical factors assumes these functions on the outer surface of the membrane.

Electron transfer-coupled H^+^ transfer was studied by Murgida and Hildebrandt (2001) via deuterium substitution. Horse heart cytochrome *c* was bound as a self-assembled monolayer (SAM) on a Ag electrode produced using different chain lengths (C_2_-C_16_) of ω-carboxyl alkanethiols. Cytochrome *c* redox reactions were performed by changing the electrical potential of the Ag electrode and monitoring changes in the Raman spectra. When the distance between cytochrome *c* and the electrode was short (C_2_; distance between cytochrome *c* and the electrode: 6.3 Å), the electron transfer rate between cytochrome *c* and the Ag electrode was slower in D_2_O (33 s^-1^) than that in H_2_O (132 s^-1^). However, there was no difference in the electron transfer rate (0.073-0.074 s^-1^) between cytochrome *c* and the Ag electrode when the distance between cytochrome *c* and the electrode was large (C_16_; distance between cytochrome *c* and the electrode: 24 Å), regardless of whether the aqueous solvent was D_2_O or H_2_O. These results indicate that the D^+^ exchange rate with amino acids in cytochrome *c* was not the rate-limiting step when the electron transfer rate between cytochrome *c* and the Ag electrode was low. H^+^-exchange-coupled electron transfer between cytochrome *c* and the Ag electrode may be the rate-limiting step if the rate of electron transfer between cytochrome *c* and the Ag electrode is high. The above-described rate-limiting H^+^/D^+^ transfer-coupled electron transfer was observed during self-assembly but not in solution. Therefore, differentiated H^+^/D^+^ transfer-coupled electron transfer can be observed only in the accumulated nanolayers of cytochromes affected by changes in the electric field (Coulomb’s force) of the Ag electrode when an electron is taken in and out concomitant with electron transfer (associated H^+^/D^+^ transfer). The fluctuating electric field may be equivariant to the local electric field strength at biological interfaces (Murgida and Hildebrandt, 2004). This finding suggests that the H^+^ transfer rate between cytochrome *c* and the H^+^ transfer network on the outer surface membrane is affected not only by electron transfer to cytochrome *c* but also by the local electric field strength on the outer surface membrane. Electron transfer and local electric field strength on the outer surface membrane may fluctuate depending on the electron transfer events in the respiratory chain.

The sulfate-reducing bacterium *D. gigas*, which reduces sulfuric acid to hydrogen sulfide, possesses large quantities of cytochrome *c*_3_, with four heme molecules in one protein (Coutinho and Xavier, 1994). The redox potential of cytochrome *c* changes depending on the pH, affecting the electric field and reducing the sequence of these hemes. Cytochrome *c*_3_ has been reported to facilitate the transfer of H^+^ to the electron transfer complex or F_1_F_o_-ATPase by cooperative H^+^/e^-^ linkage (redox-Bohr effect) (Louro et al., 1997; Messias et al., 2006). The midpoint redox potentials of heme I, heme II, heme III, and heme IV of cytochrome *c*_3_ are -306, -327, -308, and -297 mV, respectively, in solution but -332, -384, -381, and -457 mV, respectively, on the SAM on the electrode. The differences in midpoint redox potentials between the solution and the SAM follow the sequence heme IV (160 mV) > heme III (73 mV) > heme II (57 mV) > heme I (16 mV), which is consistent with the sequence for the distance between hemes and the electrode (shorter distances with larger differences in redox potentials) (Rivas et al., 2005). This finding indicates that the midpoint redox potential is affected by structural changes in the protein concomitant with changes in the electric field (Coulomb’s force). Thus, the redox potential of heme *c* is affected by localized structural changes in the vicinity of heme *c*. Changes in the intensity of the redox potential may be accounted for by the reduced distances between the hemes, and the electrode may be affected by the enhanced electric field.

To summarize the above-described phenomena regarding the behavior of redox reactions concomitant with H^+^ exchange in the aqueous phase in cytochrome *c* (or heme *c*), H^+^ transfer via the H^+^ exchange network is affected not only by the redox change in heme *c* but also by the change in the intensity of the electric field on the SAM of cytochrome *c*. In addition, the same cumulative configuration of the structure of the assembled cytochrome *c* was indispensable for these events.

## A H^+^-Condenser Produced by Cytochrome *c*-550

**FIGURE 1:**
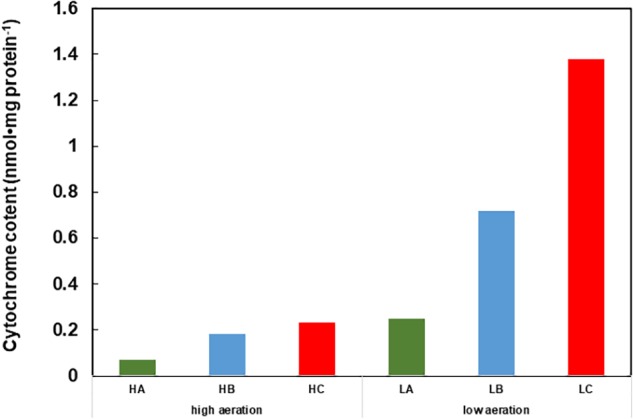
Cytochrome content of cell extracts of Gram-positive obligately alkaliphilic *Bacillus clarkii* K24-1U (grown at pH 10) under low-aeration and high-aeration conditions. HA, HB, and HC are the cytochrome *a, b*, and *c* levels, respectively, under high-aeration conditions. LA, LB, and LC are the cytochrome *a, b*, and *c* levels, respectively, under low-aeration conditions. This difference in cytochrome *c* content may indicate that cytochrome *c* has an important function under low-aeration conditions at pH 10. Low-aeration conditions were produced by using 15 L of a medium in a 20-L stainless-steel fermenter (Takasugi Seisakusho, Tokyo, Japan) with an agitation speed of 106 rpm and an air flow rate of 20 Lmin^-1^, while high-aeration conditions were produced by using 15 L of a medium in a 30-L stainless-steel fermenter (Marubishi, Tokyo, Japan) with an agitation speed of 250 rpm and an air flow rate of 20 Lmin^-1^. This figure was made according to the data of Hijikata (2004).

**FIGURE 2:**
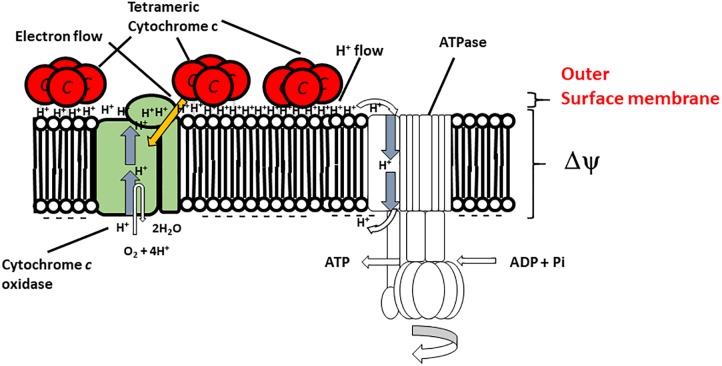
Hypothetical model of the function of membrane-bound cytochrome *c*-550 in the respiratory system of *B.* *clarkii* K24-1U. Cytochrome *c*-550 contains the specific structure Gly_22_-Asn_34_ (Asn-rich) at the N-terminal region of its sequence, which may facilitate H^+^ transfer at the interface of the outer surface membrane. The tetrameric structure is predicted to be important for enhancement of the H-bound network. The production of cytochrome *c*-550 was enhanced under low-aeration conditions. This enhanced cytochrome *c*-550 on the outer surface of the membrane led to the accumulation of electrons, H^+^ and the H^+^-condenser construct. This structure facilitates the growth of the microorganism, especially under high-pH and low-aeration conditions. This figure was produced as an original hypothetical model for this review.

In this section, we consider respiratory regulatory mechanisms based on the obtained experimental data for *B*. *clarkii* DSM 8720^T^ and *B*. *clarkii* K24-1U because both strains belong to the same species. As described above, the cytochromes *c* from alkaliphilic *Bacillus* spp. exhibited lower redox potentials (+47-+95 mV) than those from neutralophilic bacteria (+170-+230 mV) (Hicks and Krulwich, 1995; Yumoto, 2002; Goto et al., 2005) due to electron transport across the membrane between the redox center located in the outer membrane to the intramembrane side and/or transport of the heavier H^+^ from the intracellular to the extracellular side in the presence of a large ΔΨ. Although the midpoint redox potential of cytochrome *c*-550 in *B*. *clarkii* K24-1U was +83 mV, based on redox titration, the potential was even lower when determined by cyclic voltammetric measurements (Ogami et al., 2009), probably because the redox potential was affected by the electric field of the electrode during cyclic voltammetric measurement. In the abovementioned example of cytochrome *c* in the SAM, the midpoint redox potential and H^+^ transfer behavior of cytochrome *c*-550 were also affected by ΔΨ. For example, the ΔΨ of *B*. *clarkii* DSM 8720^T^ was lower under low-aeration conditions than under high-aeration conditions (Goto et al., 2016). Thus, electron transport via the respiratory chain concomitant with H^+^ transport across the membrane is relatively easy under low-aeration conditions, whereas the Δp per H^+^ is lower under low-aeration conditions than under high-aeration conditions. Under high-aeration conditions, the cytochrome *c* content of *B*. *clarkii* K24-1U (0.23 nmolmg protein^-1^) in the membrane is lower than that (1.38 nmolmg protein^-1^) under low-aeration conditions (**Figure 1**). This finding suggested that the membrane-bound cytochrome *c* in *B*. *clarkii* plays an important role under low-aeration conditions at a pH of approximately 10. Growth characteristics were studied under both conditions for alkaliphilic *B*. *clarkii* K24-1U (**Table 1**). The μ_max_ and OD_650,max_ of *B*. *clarkii* under low-aeration conditions were 0.26 h^-1^ and 0.96 (18 h), respectively, whereas the μ_max_ and OD_650,max_ under high-aeration conditions were 0.21 h^-1^ and 1.27 (12 h), respectively. These results indicated that although the growth rate and intensity of *B*. *clarkii* were greatly influenced by aeration conditions, the bacterium retained a high growth rate under low-aeration conditions. In addition, despite a culture duration of 21 h, the growth intensity remained high. This finding is quite remarkable considering the low H^+^ concentration at the outer surface membrane and the low number of terminal electron acceptors (low O_2_ concentration) in the conducting respiratory system. Therefore, it can be assumed that an increased level of membrane-bound cytochrome *c* affects H^+^ transfer at the outer surface of the membrane as follows: H^+^ condensation at the outer surface membrane via both (i) the electrical force (in reduced form) (ii) the hydrogen-bond network produced by the chemical characteristics of the protein (via Asn-rich structures). Thus, we hypothesize that the H^+^-condensation mechanism produced by cytochrome *c* in certain alkaliphilic *Bacillus* spp. plays very significant role under condition of limited aeration (**Figure 2**).

**Table 1:** Growth characteristics of the obligately alkaliphilic *Bacillus clarkii* K24-1U.

	Growth condition		
	**Low-aeration**		**High-aeration**
	
μ_max_(h^-1^)	0.26		0.21
OD_max_	0.96		1.27
Time to OD_max_ (h)	21		14
Is the growth saturated at OD_max_?	No		Yes

*Obligately alkaliphilic B. clarkii K24-1U grew at pH 10. The low aeration condition was produced by using 15 L of a medium in a 20-L stainless-steel fermenter (Takasugi Seisakusho, Tokyo, Japan) at an agitation speed of 106 rpm with an air flow rate of 20 Lmin^-*1*^, while the high-aeration condition was produced by using 15 L of a medium in a 30-L stainless-steel fermenter (Marubishi, Tokyo, Japan) at an agitation speed of 250 rpm with an air flow rate of 20 Lmin^-*1*^. This table was prepared according to the data of Hijikata (2004).*

## Soluble Form Cytochrome *c* in the Gram-Negative Alkaliphile *Pseudomonas alcaliphila* Al15-21^T^

Gram-negative alkaliphiles have been studied far less investigated than Gram-positive alkaliphiles, probably because most of the sources for alkaliphile isolation have been terrestrial samples, such as soil. The facultative alkaliphile *P. alcaliphila* AL15-21^T^ was isolated from seawater obtained from the coast of Rumoi, Hokkaido, Japan (43°56^′^N 141°38^′^E) (Yumoto et al., 2001), and the cytochromes *c* of this bacterium was studied because Gram-negative bacteria possess soluble cytochromes *c* (Matsuno et al., 2007, 2009; Matsuno and Yumoto, 2015). This property is due to the presence of periplasmic space in Gram-negative bacteria on the outer side of the membrane. *Pseudomonas* spp. belonging to the same node as *P*. *alcaliphila* (such as *P*. *mendocina* and *P*. *toyotomiensis*) in a phylogenetic tree based on the 16S rRNA gene sequence are able to grow at pH 10 (Hirota et al., 2011). This relationship between alkaline adaptation and phylogenetic position based on 16S rRNA gene sequences is similar to that observed for alkaliphilic *Bacillus* spp.

The soluble cytochrome *c* content in cells grown at pH 7-10 under low- or high-aeration conditions was estimated (Matsuno et al., 2007). The highest amount of cytochrome *c* was observed in cells grown at pH 10 under low-aeration conditions. The cytochrome *c* content in cells grown at pH 10 under low-aeration conditions (0.47 ± 0.05 nmolmg protein^-1^) was 3.6 times higher than that in cells grown at pH 7 under high-aeration conditions, which was the lowest cytochrome *c* content among the tested samples (0.13 ± 0.05 nmolmg protein^-1^) (Matsuno et al., 2009). The increased cytochrome *c* content at high pH under low-aeration conditions was similar to that observed for facultative alkaliphilic *Bacillus* spp. such as *B*. *clarkia* K24-1U (**Figure 1**; Hijikata, 2004).

The soluble fraction of *P*. *alcaliphila* AL15-21^T^ contains three types of cytochrome *c*: cytochrome *c*-552, cytochrome *c*-554, and cytochrome *c*-551. Cytochrome *c*-522 is the major soluble cytochrome *c* component, constituting 64% of the total cytochrome *c* content in *P*. *alcaliphila* AL15-21^T^ (Matsuno and Yumoto, 2015). One particular characteristic of cytochrome *c*-552 is that the resting state of this protein is similar to its fully reduced state (Matsuno et al., 2009). Thus, cytochrome *c*-552 possesses electron-retention characteristics. The molecular mass of this protein is 7.5 kDa, as determined by SDS-PAGE, which is somewhat smaller than the reported masses of cytochrome *c* proteins isolated from neutralophilic *P. aeruginosa* (9-15 kDa). A phylogenetic analysis performed using the amino acid sequence of cytochrome *c*-552 classified it as a small cytochrome *c*_5_ belonging to group 4 of class I cytochrome *c* proteins (Matsuno et al., 2007; Matsuno and Yumoto, 2015). Class I cytochromes *c* consist of six groups, and group four contains monoheme cytochromes *c* form Gram-negative bacteria such as *Pseudomonas* spp. and *Shewanella* spp. (Sone and Toh, 1994; Bertini et al., 2006; Matsuno and Yumoto, 2015). The midpoint redox potential of cytochrome *c*-552 determined by redox titration (+228 mV) was almost the same as that determined by cyclic voltammetry (+224 mV) (Matsuno et al., 2009). Cytochrome *c*-552 reacts with the terminal oxidase in the respiratory system (Matsuno et al., 2009).

The pH dependence of the cytochrome *c*-552 reduction rate was determined by estimating the reduction rate under anaerobic conditions (Matsuno et al., 2009). Cytochrome *c*-552 was fully reduced after 40 h at pH 8.5 but was fully reduced after 4 h at pH 10 in the presence of the electron mediator TMPD (*N,N,N*^′^,*N*^′^-tetramethyl-*p*-phenylenediamine). The reduction rate exhibited first-order reaction constants of 0.07 and 0.56 h^-1^ at pH 8.5 and pH 10, respectively. The oxidation rates of cytochrome *c*-552 and horse heart cytochrome *c* were estimated at pH 6-10 under ambient conditions. Cytochrome *c*-552 was oxidized very slowly at pH 8-10, with the slowest rate observed at pH 8, but was oxidized rapidly from pH 6-7. The oxidation rates of horse heart cytochrome *c* were consistently high at pH 6-10. The results demonstrated that cytochrome *c*-552 possessed distinctive electron retention characteristics. If electron-transfer-coupled H^+^ transfer (redox-Bohr effect) is possible, then cytochrome *c*-552 retains H^+^ in the periplasmic space.

**FIGURE 3:**
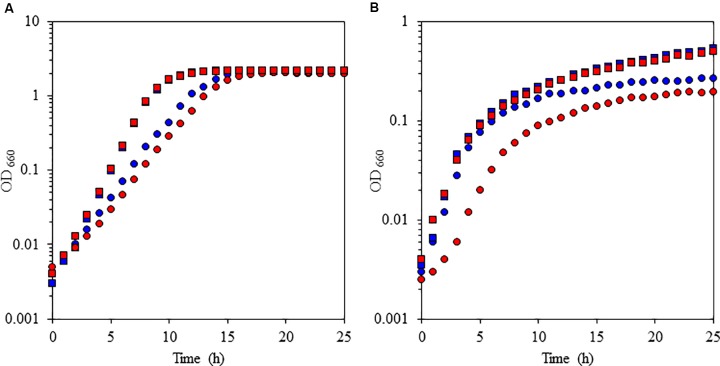
Growth characteristics of the facultative alkaliphile *Pseudomonas alcaliphila* AL15-1^T^ (wild type: blue symbols) and the cytochrome *c*-522 deletion mutant derived from the wild-type strain (mutant: red symbols) under high-aeration **(A)** and low-aeration **(B)** conditions at pH 10 (squares) and pH 7 (circles). The reproducibility of the results was confirmed by performing three independent experiments. This figure was reproduced from Matsuno and Yumoto (2015).

To understand the physiological function of *P*. *alcaliphila* AL15-21^T^ cytochrome *c*-552, an antibiotic-marker-less cytochrome *c*-552-deficient mutant was constructed to exclude the effects of antibiotics (Matsuno et al., 2011). The growth features of the wild-type *P*. *alcaliphila* AL15-21^T^ and cytochrome *c*-552 deletion mutant strains were compared at pH 10 and 7 under low- and high-aeration conditions by estimating the maximum specific growth rate (μ_max_ [h^-1^]) and maximum cell turbidity (OD_660,max_) (**Figure 3** and **Table 2**). The most significant differences in the growth parameters were observed at pH 10 under low-aeration conditions between the wild-type (μ_max_ [h^-1^] = 0.85, OD_660,max_ = 0.27) and the cytochrome *c*-522 deletion mutant (μ_max_ [h^-1^] = 0.69, OD_660,max_ = 0.20). The μ_max_ (h^-1^) of the deletion mutant was 1.5 times higher, whereas the OD_660,max_ was 26% lower than that of the wild-type strain (**Table 2**).

**Table 2:** Growth characteristics of wild-type and cytochrome *c*-552 deletion mutant strains of *P. alcaliphila* AL15-21^T^.

Growth condition								
	**Low-aeration**				**High-aeration**			
Strain	pH 10.0		pH 7.0		pH 10.0		pH 7.0	
	m_max_(h^-1^)	OD_max_	m_max_(h^-1^)	OD_max_	m_max_(h^-1^)	OD_max_	m_max_(h^-1^)	OD_max_

Wild-type	0.85	0.27	0.97	0.53	0.56	2.06	0.89	2.18
Δ*c*-552	0.69	0.20	0.92	0.50	0.49	2.06	0.72	2.17

*Wild-type: P. alcaliphila AL15-21^*T*^. Δc-552: an unmarked cytochrome c-552 deletion mutant. The mutant strain was derived from P. alcaliphila AL15-21^*T*^. μ_*max*_, maximum specific growth rate. OD_*max*_, maximum cell turbidity. μ_*max*_ and OD_*max*_ were estimated from growth at 660 nm with a model TN-2612 Biophotometer. These date were reproduced from Matsuno and Yumoto (2015)*.

**FIGURE 4:**
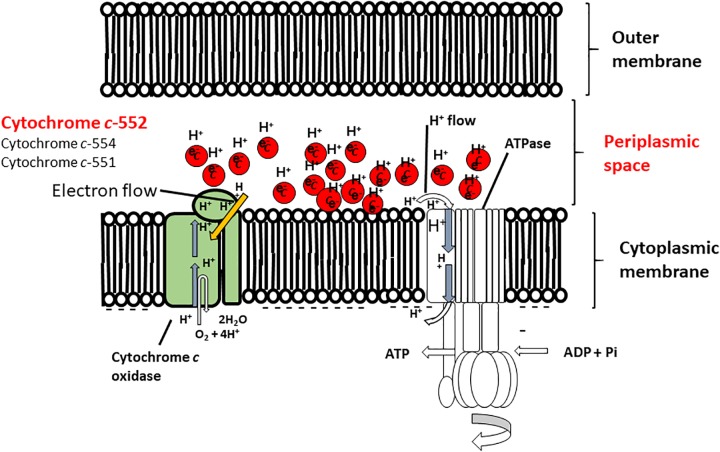
Hypothetical model of the function of cytochrome *c*-552 in the respiratory system of the Gram-negative facultative alkaliphilic *P. alcaliphila* AL15-1^T^. This bacterium possesses cytochrome *c*-552 (monoheme), cytochrome *c*-554 (monoheme), and cytochrome 551 (diheme), all of which tend to retain the reduced state. Cytochrome *c*-552 is a major soluble cytochrome *c* constituent in the periplasmic space. The features of cytochrome *c*-552 cause in high electron retention and attract H^+^, and the development of electron- and H^+^ -retention conditions occurs in the periplasmic space of microorganisms concomitant with an abundance of this cytochrome *c* under low-aeration conditions at pH 10. Thus, Gram-negative *P. alcaliphila* AL15-1^T^ expresses an electron and H^+^ condenser in the periplasmic space. This figure was reproduced based on a hypothetical model presented in Matsuno and Yumoto (2015).

The oxygen consumption rates of cell suspensions of the wild-type strain and the cytochrome *c*-522 deletion mutant of *P*. *alcaliphila* AL15-21^T^ were assessed under different conditions (i.e., pH 7 or pH 10; high or low aeration) (Matsuno et al., 2011). The oxygen consumption rates in the cytochrome *c*-522 deletion mutant cells under low- and high-aeration conditions increased by 12 and 17%, respectively, compared to those in the wild type. This finding indicates that cytochrome *c*-552 hinders the direct electron transfer in the respiratory chain. A possible role of this protein might be the formation of an electron bypass to construct an electron reservoir in the periplasmic space. This hypothesis is consistent with the finding that cytochrome *c*-552 has strong electron retention ability at a high pH values (**Figure 4**).

## Conclusion and Perspectives

Bacteria utilize several strategies to adapt to high pH, avoid OH^-^ and manage scarce H^+^ resources via secondary cell wall components, the Na^+^-based transportation system and flagellar rotation. However, for the respiratory system, the management of energy production under one-thousandth of the ambient concentrations of H^+^ is difficult. In the case of alkaliphilic *Bacillus* spp., a large ΔΨ is indispensable for adaptation at high pH. This high value not only compensates for the deficient ΔpH but also attracts H^+^ moieties that are translocated by the respiratory chain and affect the redox potential of the cytochrome *c* bound to the outer surface membrane. However, although we have not determined the ΔΨ of alkaliphilic *Pseudomonas* spp., ΔΨ is not believed to greatly influence the cytochrome *c* in the periplasmic space. This difference may be attributable to differences in the proteins present in the periplasmic space in Gram-negative *Pseudomonas* spp. Thus, the effect of ΔΨ is assumed to encompass the outer surface membrane, although this parameter hardly affects the entire periplasmic space. The main region involved in alkaline adaptation in alkaliphilic *Bacillus* spp. is the outer surface membrane, comprising the ΔΨ (physical parameter) and proteins (associated with the acidic nature and retention and transportation of H^+^ and electrons) located at the outer surface membrane. In contrast, the main region involved in adaptation in alkaliphilic *Pseudomonas* spp. is the periplasmic space and the corresponding localized proteins (associated with the acidic nature and retention and transportation of H^+^ and electrons).

Cytochrome *c* is a well-known electron carrier in the respiratory chain. However, as described above, despite several reports regarding the H^+^-transferring characteristics of cytochrome *c*, it is difficult to conclude that these characteristics are well understood. Studies of cytochrome *c* in alkaliphilic bacteria have shown that not only the H^+^-transferring characteristics but also the H^+^-retention features of cytochrome *c* are important for energy production, especially under low-aeration conditions. We propose that cytochrome *c* participates in the “H^+^ capacitor mechanism” as an energy production strategy under low-aeration and alkaline conditions. Surprisingly, although the cell surface structure is completely different between Gram-positive and Gram-negative bacteria, the H^+^ and electron retention characteristics of cytochrome *c* are important. However, there are several differences (the charge in redox potential depends on ΔΨ, the value of the redox potential and electron retention ability) among cytochrome *c* proteins. These changes may be attributable to strategic differences in the reaction site, which is an interfacial surface (outer surface membrane) or space (periplasmic space). As described above, cytochrome *c* has multiple functions (electron carrier or reservoir, H^+^ carrier or reservoir and acidic nature). However, pronounced expression of cytochrome *c* is not considered indispensable for adaptation under alkaline conditions because some alkaliphilic *Bacillus* spp. do not express large amounts of cytochrome *c*. According to the experimental results described above, alkaliphiles that express large amounts of cytochrome *c* likely exhibit superior growth features at a high pH at low-oxygen concentrations.

## Author Contributions

KoY, NI, HiM, KaY, and IY designed the research. TM, TG, SO, and HaM performed the research. KaY and IY analyzed the data and wrote the paper.

## Conflict of Interest Statement

The authors declare that the research was conducted in the absence of any commercial or financial relationships that could be construed as a potential conflict of interest.
